# High level expression of *Acidothermus cellulolyticus *β-1, 4-endoglucanase in transgenic rice enhances the hydrolysis of its straw by cultured cow gastric fluid

**DOI:** 10.1186/1754-6834-4-58

**Published:** 2011-12-10

**Authors:** Hong Li Chou, Ziyu Dai, Chia Wen Hsieh, Maurice SB Ku

**Affiliations:** 1 Institute of Bioagricultural Science, National Chiayi University, Chiayi, 60004 Taiwan; 2 Fungal Biotechnology Team, Chemical and Biological Processing Development Group, Pacific Northwest National Laboratory, Richland, WA, 99352, USA; 3 Departmet of Microbiology, Immunology and Biopharmaceuticals, National Chiayi University, Chiayi, 60004 Taiwan; 4 School of Biological Sciences, Washington State University, Pullman, WA 99164-4238, USA

## Abstract

**Background:**

Large-scale production of effective cellulose hydrolytic enzymes is the key to the bioconversion of agricultural residues to ethanol. The goal of this study was to develop a rice plant as a bioreactor for the large-scale production of cellulose hydrolytic enzymes via genetic transformation, and to simultaneously improve rice straw as an efficient biomass feedstock for conversion of cellulose to glucose.

**Results:**

In this study, the cellulose hydrolytic enzyme β-1, 4-endoglucanase (*E1*) gene, from the thermophilic bacterium *Acidothermus cellulolyticus*, was overexpressed in rice through *Agrobacterium*-mediated transformation. The expression of the bacterial *E1 *gene in rice was driven by the constitutive Mac promoter, a hybrid promoter of Ti plasmid mannopine synthetase promoter and cauliflower mosaic virus 35S promoter enhancer, with the signal peptide of tobacco pathogenesis-related protein for targeting the E1 protein to the apoplastic compartment for storage. A total of 52 transgenic rice plants from six independent lines expressing the bacterial E1 enzyme were obtained that expressed the gene at high levels without severely impairing plant growth and development. However, some transgenic plants exhibited a shorter stature and flowered earlier than the wild type plants. The E1 specific activities in the leaves of the highest expressing transgenic rice lines were about 20-fold higher than those of various transgenic plants obtained in previous studies and the protein amounts accounted for up to 6.1% of the total leaf soluble protein. A zymogram and temperature-dependent activity analyses demonstrated the thermostability of the E1 enzyme and its substrate specificity against cellulose, and a simple heat treatment can be used to purify the protein. In addition, hydrolysis of transgenic rice straw with cultured cow gastric fluid for one hour at 39°C and another hour at 81°C yielded 43% more reducing sugars than wild type rice straw.

**Conclusion:**

Taken together, these data suggest that transgenic rice can effectively serve as a bioreactor for the large-scale production of active, thermostable cellulose hydrolytic enzymes. As a feedstock, direct expression of large amount of cellulases in transgenic rice may also facilitate saccharification of cellulose in rice straw and significantly reduce the costs for hydrolytic enzymes.

## Background

In facing increasing demands for energy and depleting fossil oil reserve, the adoption of alternative, renewable energy is imperative. In the past decade, utilization of biomass for fuel production has been considered not only practical but also extremely vital with respect to the development of sustainable energy [1-3]. Biofuels converted from biomass, considered renewable, environment friendly and carbon neutral, will serve a more important function in the days to come. Although the production of biofuels from starch, sugar or oil from traditional food crops, such as corn, sugarcane, soybean and canola, is relatively simple, it competes with human beings and animals for foods and requires a high energy input for cultivation of these crops. Use of lignocellulosic crops or agricultural residues, such as rice straw or corn stover, for ethanol production is not only economical (high energy output to input ratio) but also environment friendly (that is, carbon neutral and with the emission of less toxic pollutants) and will curtail our reliance on fossil oil and help prevent global warming [4].

Lignocellulose is the major polysaccharide component of global plant mass, which consists of hemicellulose, lignin and cellulose, a polymer of thousands of 1, 4-β-linked unions of D-glucose [5,6]. The polymer is arranged in collinear, semi-crystalline bundles and it comprises up to 45% of the dry weight of plant biomass, which is a potentially inexpensive, renewable source of fermentable glucose [7,8]. Rice is one of the most important food resources in the world, and global rice production has risen steadily from about 200 million metric tons in 1960 to over 660 million metric tons in 2009 [9]. At the same time, about 800 million metric tons of rice straw is also produced annually, which is normally burned or decayed in the field, producing more pollutants and greenhouse gases (for example, methane). Thus, developing rice as a dual-functional crop for solving both the immediate food and energy crisis issues could pave the road toward the successful development of sustainable energy and the prevention of possible pollution from agricultural wastes. However, the conversion of the polysaccharide component of lignocellulose into ethanol for use as an alternative transportation fuel and other useful chemicals requires a series of complete pretreatment and hydrolysis procedures [2,6,10]. The complete hydrolysis of cellulose (saccharification) requires at least three different hydrolytic enzymes, including β-1,4-endoglucanse (EC 3.2.1.4), β-1, 4-exoglucanse (EC 3.2.1.91), and β-D-glucosidase (EC 3.2.1.21) [2,10,11].

Large-scale production of effective cellulose hydrolytic enzymes is the key to the bioconversion of lignocellulose to fermentative sugars for biofuel and chemical production. Nowadays, production costs and the performance of hydrolytic enzymes from bacterial and fungal sources for cellulosic ethanol production remain the major obstacles because the essential nutrients and the maintenance of optimal conditions are very expensive and laborious [3,12]. Nevertheless, there has been significant progress in bringing effective and inexpensive means of cellulase production from a large scale bioreactor, in the conversion of low value lignocellulosic material into a cost effective fermentative sugars for producing biofuels. Replacing microorganisms with transgenic plants for the production of cellulose hydrolytic enzymes will significantly reduce the production costs. In other words, manufacturing heterologous cellulases in plants (especially in energy crops) using genetic engineering would lower the expense associated with enzyme production and the amount of enzyme loading required during saccharification [11,13]. So far, a number of studies have attempted to express cell wall-related degrading enzymes in transgenic plants, such as β-glucosidases [14,15], ferulic acid esterases [16,17], xylanases [18], cellobiohydrase [19,20] and endoglucanase [7,21-26]. However, there may be a trade off between expressing foreign cellulases in transgenic plants, obstructing their normal growth [11] and low level expression [27,28]. Hence, it is essential to design a proper expression system for the production of plants expressing microbial cellulases, without perturbing their growth and development. This system needs to include a strong promoter with a high-level transcription capacity and an appropriate signal peptide to target the foreign protein into a suitable compartment for high level accumulation [21].

*A. cellulolyticus *E1 endoglucanase is a well-known thermostable enzyme, which exhibits low activity at ambient temperatures [21,22,29]. This desirable E1 feature might have less deleterious effects on the growth and development of transgenic plants under normal growth conditions. Transgenic expression of thermostable E1 endoglucanase has been examined in *Arabidopsis*, potato, maize, rice and tobacco [7,21-25], with the foreign protein accounting for up to 0.01% to approximately 25.7% of the total soluble protein, depending on the host plant and its tissue. In addition, when expressed in plants, the E1 enzyme exhibits high temperature stability (optimum 81°C) and relatively low activity at general room temperatures [21].

The major objective of this study was to express and accumulate *A. cellulolyticus *E1 in the apoplastic compartment of rice in large quantities. The expression of the bacterial *E1 *gene in rice was under the control of the strong constitutive Mac promoter, which has been previously shown to have three- to five-fold higher strength than the cauliflower mosaic virus 35S promoter [30], and targeted the E1 protein to the apoplast compartment for higher accumulation. We report that high expression levels of active E1 enzyme were achieved in transgenic rice plants, which accumulated up to 6.1% of total leaf soluble protein. Moreover, this thermostable enzyme can be purified from crude extract by a simple heat treatment. In addition, digestion of transgenic rice straw with cultured cow gastric fluid (CGF) for one hour at 39°C and another hour at 81°C yielded 43% more reducing sugars than the wild type rice straw. Thus, the present study demonstrates the technical feasibility and great potential to reduce the cost of producing cell wall-degrading enzymes from transgenic crops.

## Results

### Rice transformation and transgene confirmation

**Figure 1:**
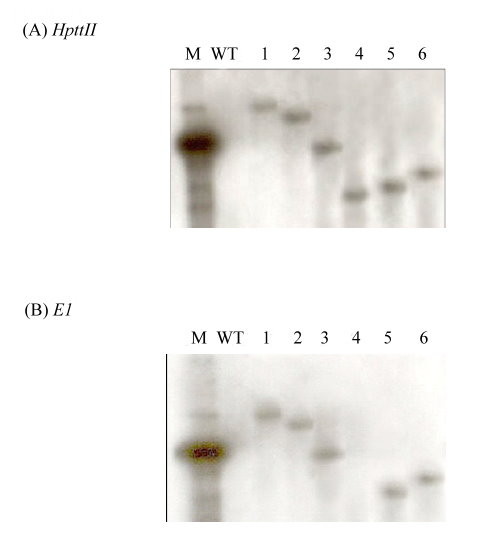
**Southern blot analysis of *HptII *and *E1 *genes**. Genomic DNA was extracted from six independent T0 transgenic lines (#1 to #6) and wild type (WT) rice plants, digested with *Hind*III, separated by agarose gel electrophoresis, transferred to nylon membrane and hybridized with **(A) ***HptII *probe or **(B) ***E1 *probe. Note that *E1 *gene was not detected in transgenic line #4 and wild type rice plants.

The *A. cellulolyticus E1 *and hygromycin phosphotransferase II (*HptII*) genes in the *p*1500 binary vector were successfully introduced into rice genome by *Agrobacterium*-mediated transformation. Fourteen independent T0 transgenic lines with a total of 84 transgenic plants were obtained for an E1 activity assay. Most transgenic plants exhibited a normal phenotype and fertility. Noticeably, some transgenic rice plants with high levels of expression tended to have a shorter stature and flowered one week earlier than the wild type rice plants. One rice plant from each line was chosen to obtain self-pollinated seeds. Sixty T1 seeds from each line were germinated on 1/2 Murashige and Skoog medium containing 50 mg/L hygromycin, from which six lines exhibited a germination ratio of approximately three to one (48:12, 45:15, 43:17, 47:13, 44:16 and 47:13), indicating one copy of *HptII *gene insertion in these lines. For each line, all hygromycin-resistant plants were transplanted to soil and grown in the greenhouse for further characterization in terms of *E1*expression. Southern blotting analysis with the T0 plants of the six transgenic lines that showed a 3:1 germination ratio of T1 seeds in hygromycin medium confirmed the single-copy insertion in all six transgenic lines, except transgenic line #4 that only contained the *HptII *gene (Figure 1).

### *E1 *gene expression in transgenic rice

**Figure 2:**
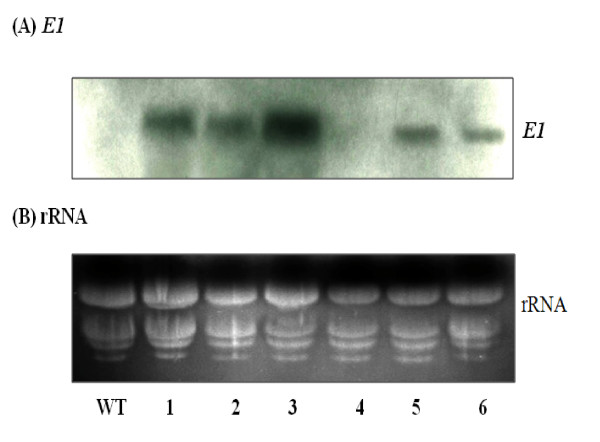
**Northern blot analysis of *E1 *gene transcript in the leaves**. Twenty-five micrograms of total RNA, extracted from the leaves of six independent T0 transgenic lines (#1 to #6) and wild type (WT) rice plants, was loaded into each lane, transferred to nylon membrane after electrophoresis and probed with **(A) ***E1 *DNA or **(B) **stained for rRNA by EtBr. Note that no *E1 *gene transcript was detected in transgenic line #4 and wild type plants.

**Figure 3:**
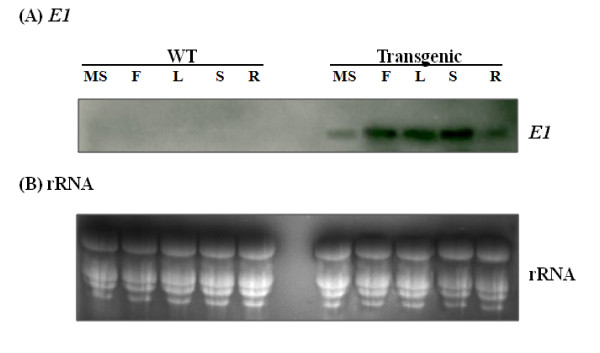
**Tissue-specific expression of *E1 *gene, as analyzed by northern blot analysis**. Twenty-five micrograms of total RNA, extracted from mature seed (MS), green floret (F), leaf (L), stem (S), and root (R) of T0 transgenic line #3-5 (one of the T0 plants of transgenic line #3) and wild type (WT) rice plants, was loaded into each lane, transferred to nylon membrane after electrophoresis and probed with **(A) ***E1 *DNA or **(B) **stained for rRNA by EtBr.

Northern blot analysis of the *E1 *transcript, of a predicted 1.75 kb mRNA, was also detected in leaf tissues of all transgenic lines, except line #4 and the wild type plant (Figure 2). These results demonstrate that the integrated *E1 *gene from *A*. *cellulolyticus *can be properly transcribed in rice. Although the Mac promoter is expected to drive constitutive expression of *E1 *gene in transgenic plants, the gene was expressed in a somewhat organ-specific manner, with the highest levels of transcript being detected in the leaf and the lowest levels in the root (Figure 3).

**Figure 4:**
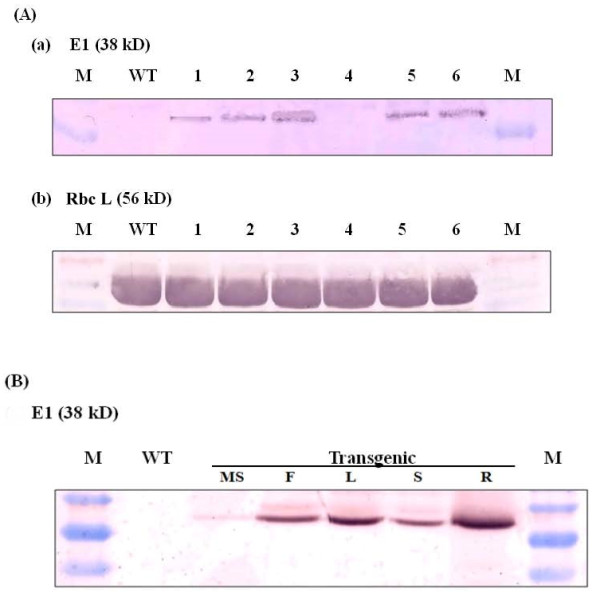
**Western immunoblot analysis of E1 and Rubisco large subunit proteins in the leaf and different organs**. Total soluble protein was extracted from the leaves of six independent T0 transgenic lines (#1 to #6) and wild type (WT) rice plants and from the mature seed (MS), green floret (F), leaf (L), stem (S) and root (R) of T0 transgenic line #3-5, separated by SDS-PAGE, transferred to polyvinylidene fluoride membrane and probed with polyclonal antibodies against **(A) **E1 or **(B) **Rubisco large subunit. Twenty micrograms of protein was loaded per lane. Note that E1 protein was not detected in the leaves of transgenic line #4 and wild type rice plants.

The level of E1 protein accumulated in leaves among different transgenic lines and various organs was assessed by western blotting analysis (Figure 4). Rabbit antibody raised against E1 enzyme detected the protein in the leaves of five of the six transgenic rice lines, with the molecular mass of approximately 38 kDa, which corresponds to the catalytic domain of endoglucanase E1 (Figure 4A). The highest level of E1 accumulation was found in transgenic line #3, while no E1 protein was detected in the wild type rice plant and transgenic line #4. Furthermore, a substantial amount of E1 protein was accumulated in green floret, leaf, stem and root when compared on a protein basis, with a small amount accumulated in the mature seed (Figure 4B).

### Thermostability of the E1 protein and purification

**Figure 5:**
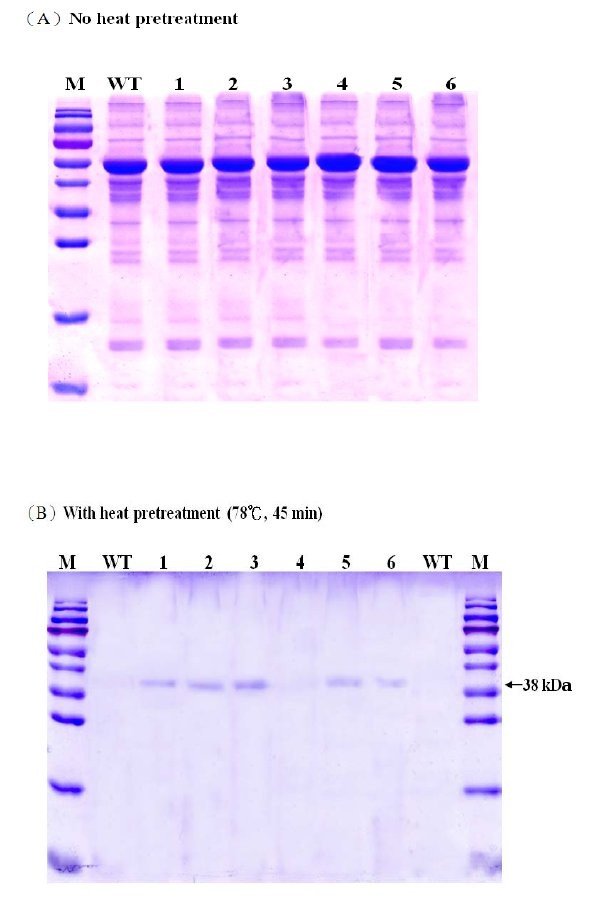
**SDS-PAGE analysis of leaf soluble protein with or without prior heat pretreatment**. Total leaf soluble protein was extracted from newly matured leaves of six independent T0 transgenic (#1 to #6) and wild type (WT) rice plants **(A) **without or **(B) **with prior heat treatment at 78°C for 45 min before electrophoretic separation by SDS-PAGE. Twenty micrograms of protein was loaded per lane. M: protein standards. After electrophoresis, gels were stained with Coomassie Brilliant Blue R-250.

The profiles of total leaf soluble protein for the wild type and six transgenic lines were examined by SDS-PAGE and staining with Coomassie Brilliant Blue (Figure 5). Figure 5A shows the profiles of the soluble protein after being boiled for two minutes in a buffer containing β-mercaptoethanol and SDS. As anticipated, after separation by SDS-PAGE the most abundant proteins in the leaf samples were Rubisco large (56 kDa) and small (14 kDa) subunits. As E1 is a thermostable enzyme, it should be more stable than the majority of other plant proteins under heat treatment [7,25,26,29]. When the leaf protein extracts of transgenic lines #1, #2, #3, #5 and #6, which contained the *A. cellulolyticus E1 *gene, were subjected to a heat pretreatment and centrifugation, the 38 kDa E1 polypeptide corresponding to the E1 catalytic domain was the major protein left in the samples (Figure 5B). No detectable E1 protein was left in the heat-treated leaf protein samples of the wild type plant or transgenic line #4. Consequently, based on this feature, E1 protein can be easily purified from the tissues of transgenic rice plants by this simple and rapid procedure.

### Enzymatic activity and zymogram analysis of E1 protein

**Figure 6:**
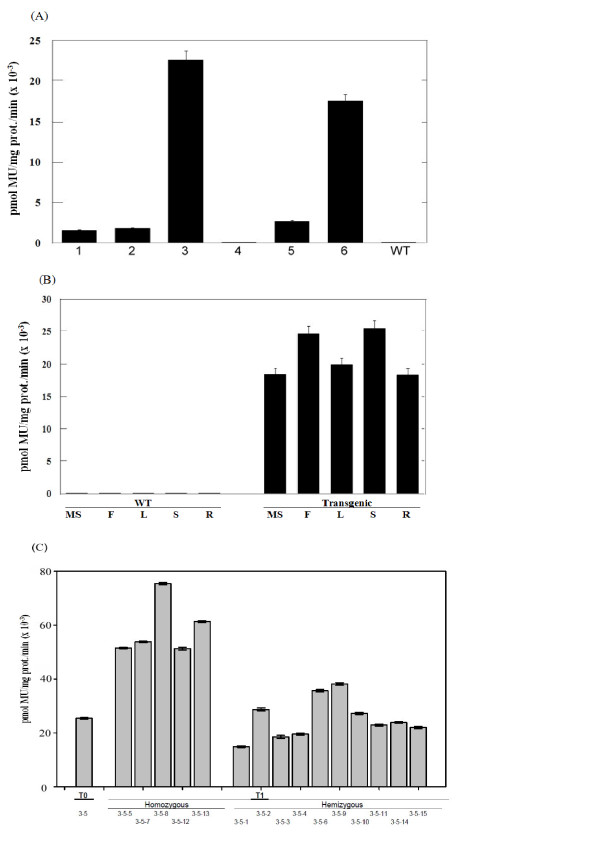
**Specific activities of E1 enzyme in different organs**. Specific activities of **(A) **E1 in the leaves of six independent T0 transgenic line (#1 to #6) and wild type (WT) rice plants, **(B) **in the mature seed (MS), green floret (F), leaf (L), stem (S) and root (R) of T0 transgenic line #3-5, and (C) in the leaves of some T1 plants of transgenic line #3-5. Enzyme activity was assayed using a fluorescence spectrophotometer by its ability to cleave 4-methylumbelliferyl β-D-cellobioside to produce the fluorophore, 4-methylumbelliferne with peak excitation wavelength at 365 nm and peak fluorescence at 455 nm. Data presented were means ± standard error (bar) from three to four replicates of measurement. The activity was measured at 65°C. Note both transgenic line #4 and wild type (WT) had minimal activities.

The enzymatic activities of E1 in the leaf extracts of different primary (T0) rice transformants are shown in Figure 6A. A wide range of activity was detected among the five transgenic lines containing the *E1 *gene. As expected, both the wild type plant and transgenic line #4 had very little endoglucanase activity. In accordance with the results of northern blot and western immunoblot analyses, transgenic line #3 exhibited the highest E1 activity of over 22,000 pmol MU/mg protein/min, while line #6 also yielded very high E1 activities (17,500 pmol MU/mg protein/min). The E1 activities of the other three lines were only about one tenth the levels of transgenic lines #3 and #6. The activity of *A. cellulolyticus *E1 in different plant tissues was also examined and ranged from 18,000 pmol MU/mg protein/min to 25, 000 pmol MU/mg protein/min (Figure 6B). Furthermore, after germination of seeds derived from the self-pollination of T0 transgenic plants on hygromycin-containing medium, the E1 activity was examined in the leaves of 15 randomly selected T1 plants from each line. The activity data for transgenic line #3-5 (one of the T0 plants of transgenic line #3 that possesses the highest enzyme activity) and its own next generation T1 plants (#3-5-1 to #3-5-15) can be grouped into two classes based on the E1 activity phenotype: 10 plants with E1 activities similar to that of T0 plants (hemizygous), and five plants with E1 activities about twice that of T0 plants (homozygous) (Figure 6C). The data for the other five lines also showed a similar segregation pattern (data not shown). Thus, the T1 plants segregated into a 2:1 ratio for E1 activity phenotype, in agreement with the notion that the *A. cellulolyticus E1 *gene was present in the genome of transgenic plants in one copy (Figure 1). The E1 specific activities in the leaves of homozygous plants of transgenic line #3 ranged from 55,000 pmol MU/mg protein/min to 79,000 pmol MU/mg protein/min.

**Figure 7:**
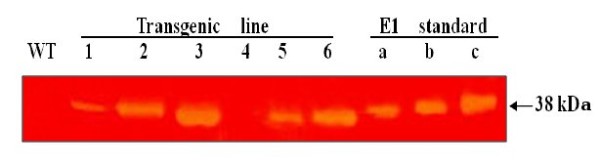
**Zymogram analysis of E1 enzyme activity in the leaves**. Total soluble protein was extracted from the leaves of six independent T0 transgenic lines (#1 to #6) and wild type (WT) rice plants and separated by SDS-PAGE in gel containing 0.1% carboxymethyl cellulose. After electrophoretic separation (20 μg/lane), the gel was washed and E1 activity stained *in situ *with Congo Red. The size of the clear zone indicates the degree of activity. Note that no E1 activity was detected in transgenic #4 and wild type plants. Purified E1 protein was obtained by heating (78°C) the leaf soluble protein extract of T0 transgenic line #3-5 for 45 min, and purified E1 protein: **(A) **1 μg; **(B) **2 μg; **(C) **3 μg.

A zymogram with native PAGE was applied to test the leaf E1 activity by digesting carboxymethyl cellulose (CMC) *in situ*. As shown in Figure 7, a single clearance band was detected for transgenic line #1, #2, #3, #5 and #6, but not in the wild type and transgenic line #4, clearly indicating the digestion of CMC by the 38 kDa endoglucanase after incubation of the gel at 65°C for 30 min. Again, transgenic lines #3 and #6 also showed the highest CMC digestion activities; the related amounts of E1 protein ranged from 0.8% in transgenic line #1 to 6.1% of the total leaf soluble protein in transgenic line #3.

### Enzymatic hydrolysis of rice straw with cultured CGF

**Figure 8:**
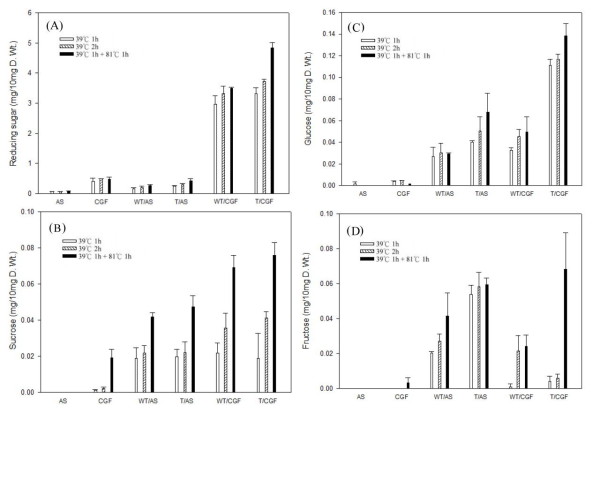
**Release of sugars from rice straw during digestion with artificial saliva or cultured cow gastric fluid**. The contents of **(A) **reducing sugars, **(B) **sucrose, **(C) **glucose and **(D) **fructose from the straws of wild type (WT) and homozygous E1 transgenic rice plants (derived from line #3-5) after incubation with AS or cultured CGF at 39°C for 1 h, 39°C for 2 h, or 39°C for 1 h plus 81°C for 1 h. WT/AS: WT rice straw incubated with AS; T/AS: transgenic rice straw incubated with AS; WT/CGF: WT rice straw incubated with CGF; T/CGF: transgenic rice straw incubated with CGF. Rice straw was air dried under the sun and stored at room temperature for two years. Data presented represent the means ± standard error of three replicates of measurement. AS: artificial saliva; CGF: cow gastric fluid; T: transgenic; WT: wildtype.

To test if transgenic rice straw expressing the bacterial E1 protein could be more efficient in producing reducing sugars than non-transgenic one, pulverized rice straw was subjected to enzymatic hydrolysis by concentrated cultured CGF containing xylanase. The amounts of reducing sugars, sucrose, glucose and fructose in the digestion media were monitored during digestion under different temperature conditions. Low amounts of these sugars were present in the air-dried rice straw samples of both wild type and E1 transgenic plants (homozygous plants of transgenic line #3-5), artificial saliva (AS) and CGF (Figure 8). The AS was employed in an attempt to maintain the chemical compositions that approximate those of the CGF environment except for the hydrolytic enzymes. After incubation with AS at 39°C for 1 h, 39°C for 2 h, or 39°C for 1 h plus 81°C for 1 h, the amounts of the reducing sugars and non-reducing sugar (sucrose) released from both transgenic and non-transgenic wild type rice straw were low. The results indicate that the air dried rice straw from both genotypes contained low levels of reducing sugars and sucrose. There were increases in the amounts of sucrose, glucose and fructose after 1 h digestion at 39°C, but the overall contents of these sugars remained low (0.02 mg/10 mg to 0.06 mg/10 mg dry weight). However, there were large increases in the amount of these sugars, except for fructose, from both wild type and transgenic rice straw samples at 39°C for 1 h and 39°C for 2 h when digested with CGF, which had xylanase activity; and the increases were most significant for transgenic rice straw, especially under the digestion condition of 39°C for 1 h plus 81°C for 1 h. After digestion at 39°C for 1 h, the additional 1 h digestion at 39°C only gave rise to a small increase in these sugars, while the most significant increases in reducing sugars, sucrose and glucose were found in transgenic rice straw after additional digestion at 81°C (an optimal temperature for E1 enzyme) for 1 h. For example, for wild type rice straw the reducing sugar, sucrose and glucose contents (dry weight) increased from 3.32 mg/10 mg to 3.51 mg/10 mg (net increase 0.19 mg/10 mg), 0.036 mg/10 mg to 0.069 mg/10 mg (net increase 0.033 mg/10 mg) and 0.045 mg/10 mg to 0.050 mg/10 mg (net increase 0.005 mg/10 mg), respectively. On the other hand, for E1 transgenic rice straw, the corresponding increases for these sugars (dry weight) were from 3.73 mg/10 mg to 4.83 mg/10 mg (net increase 1.10 mg/10 mg), 0.041 mg/10 mg to 0.076 mg/10 mg (net increase 0.035 mg/10 mg) and 0.117 mg/10 mg to 0.139 mg/10 mg (net increase 0.022 mg/10 mg), respectively. Thus, relative to wild type rice straw, E1 transgenic rice straw released additional reducing sugars (0.19 mg/10 mg versus 1.10 mg/10 mg dry weight) and glucose (0.005 mg/10 mg versus 0.022 mg/10 mg dry weight) during this high temperature digestion, presumably due to the presence of E1 protein in its straw. Apparently, high temperature treatment increased the efficiency of cellulose hydrolysis through the action of the thermophilic E1 in the transgenic straw.

The presence of free sucrose in the straw could have contributed to the reducing sugars released during the hydrolysis experiment. However, our data showed that air-dried straw from both transgenic and wild type rice plants contained low amounts of reducing sugar, sucrose, glucose and fructose. This is consistent with data from a recent study by Park *et al*. [31], which showed that air-dried rice straw contained minimal amounts of sucrose and free glucose and fructose, presumably due to continued respiration and bacterial contamination during drying. Thus, our results demonstrate that, with a starting straw of 10 mg dry matter, after 2 h incubation (at 39°C for 1 h plus 81°C for 1 h) approximately 4.83 mg and 3.52 mg reducing sugars were released from the transgenic and wild type straw, respectively. After correcting for the background amount of reducing sugars in the CGF (0.48 mg/10 mg) this 1.31 mg of additional reducing sugars released through the action of *A. cellulolyticus *E1 in the transgenic rice straw represented a 43% increase. There were no corresponding increases in glucose and fructose, which may be related to the fact that digestion of cellulose by endoglucanase mainly releases cellobiose.

## Discussion

Global rice production had reached 660 million dry metric tons in 2009, along with 800 million metric tons of straw, and continues to grow at a steady rate [32]. Rice straw contains a high cellulose content (approximately 45%), and is a suitable resource for the large-scale production of biofuels to replace fossil fuels and to reduce environmental pollution caused by agricultural wastes and burning of fossil fuels [33]. Use of cell wall-degrading enzymes from microorganisms for the conversion of cellulosic material to sugars is a limiting step at present. Thus, large-scale production of effective cellulose hydrolytic enzymes is key to the biofuels industry. The expression of heterologous proteins in transgenic plants is an established technology. Consequently, the expression of foreign proteins has been successfully applied in plant systems at various levels, including industrially useful enzymes, viral proteins, pharmaceutical proteins, polypeptides (including antibodies) and various structural proteins [34-37]. The factors influencing the level of accumulation for each of these protein classes vary. Although success depends on the characteristics of the individual protein, protein accumulation has been particularly successful when targeted to the cell wall (apoplast compartment), vacuole or endoplasmic reticulum [11,38].

This study demonstrated that the *E1 *gene of *A. cellulolyticus *integrated into the genome of transgenic rice plants (Figure 1) can be properly transcribed (Figures 2 and 3) and translated into an active enzyme and accumulated at high levels (Figures 4, 5, 6 and 7). We noticed that some of the transgenic rice plants with high E1 activities exhibited a short stature and flowered earlier than wild type plants. However, in a previous study, transgenic rice overexpressing *A. cellulolyticus *did not show any deleterious effect on plant growth and development [24]. The differences in E1 effect on rice plant growth and development between the present study and that of Oraby *et al*. [24] could in part stem from expression level. The highest expression level achieved in our transgenic lines was 6.1% of the total leaf soluble protein, higher than that reported by Oraby *et al*. [24]. The controversy regarding the effects of E1 on plant growth and development has been reported previously [11,24]. It also could be related to the difference in the primary structure of cell walls and its composition between monocot (rice) and dicot (tobacco) plants [39,40] or the position effects of *E1 *transgene insertion in the genome of transgenic plants. Further study is needed to address the effect of growth temperature on the growth and development of E1 transgenic rice. Most importantly, the E1 protein produced in rice retains its thermostability and can be purified by a simple heat treatment (Figure 5). Also significant is the fact that it remains active in tissues after a long period of storage at room temperature and the presence of E1 in the rice straw increases its hydrolytic efficiency (Figure 8).

The *A. cellulolyticus *E1 activity could be detected in all tissues of all five transgenic lines. Its activities in the leaves ranged from 60,000 pmol MU/mg protein/min to 79,000 pmol MU/mg protein/min in the homozygous plants derived from the line with the highest expression level (Figure 4). The large (ten-fold) variation in expression among the five transgenic lines containing *E1 *(Figure 4) was presumably a position effect, as all transgenic lines had one copy of insertion (Figure 1). This highlights the importance of generating as many transgenic plants as possible for selecting high expression lines with a normal phenotype. Transformation of rice with the E1 expression vector under the control of a constitutive Mac promoter [30] with the tobacco pathogenesis related signal peptide [21] allowed the heterologous E1 protein to be secreted to and accumulated in the apoplastic compartment at high levels. This is consistent with previous studies reporting that the apoplast can serve as a storage site for large quantities of functional foreign proteins [7]. Based on the zymogram PAGE (Figure 7) and western blot analysis data (Figure 4), E1 protein in all tissue extracts of the transgenic plants was partially degraded to its catalytic domain polypeptide of 38 kDa (Figure 5), in agreement with the observation of previous studies [7,23,41,42]. Obviously, the degraded protein still retains its catalytic activity. The partial degradation of E1 protein may be due to the sensitivity of the cellulose-binding domain to proteases in plant extracts. Further study will be necessary to illustrate the E1 protein degradation pattern in plant tissues.

Although the Mac promoter is known to drive constitutive expression of transgenes in plants and the *E1 *transcript was detected in all tissues, including the mature seed, floret, leaf, stem and root of transgenic rice (Figures 2,3,4B and 6B), much higher expression levels were found in green tissues, such as leaf, floret and stem (Figure 3). However, a relative large amount of E1 protein was detected in the root soluble protein sample in the western blot analysis (Figure 4B), but a relatively low E1 specific enzyme activity was found in the enzyme activity assay (Figure 6B). This is due to the fact that an equal amount of the total soluble protein for each sample was used in both analyses for comparison but root extract contains a relative low amount of other soluble protein and some potential inhibitors to the activity of E1. The highest amount of E1 protein accumulation, in the leaves of our transgenic rice plants, was up to 6.1% of the total leaf soluble protein (Figure 7), higher than the 4.9% in the transgenic rice plants reported by Oraby *et al*. [24], in which the cauliflower mosaic virus 35S promoter was used to drive the expression of the bacterial *E1 *gene in rice. Thus, the Mac promoter may be more effective for E1 protein production. However, the position of the gene insertion into the genome apparently has a strong influence on transgene expression. With a conservative estimate of ten metric tons of rice straw produced per hectare per year and a 5% E1 protein content in the tissues, 30 kg of very pure E1 protein can be produced annually from a hectare of padding field.

The high thermostability and protease-resistance of *A. cellulolyticus *E1 protein allows the enzyme to remain active in the tissues for a long period of time. Most importantly, as demonstrated in the digestion experiment with cultured CGF (Figure 8), direct expression of *A. cellulolyticus *E1 in the transgenic rice straw increases the hydrolytic efficiency of cellulose during saccharification, which can reduce the amount of hydrolytic enzyme needed for the conversion of cellulosic biomass to fermentative sugars. A recent study with transgenic tobacco and maize suggests that the expression of *A. cellulolyticus *E1 during cell wall construction may alter the inherent recalcitrance of the cell wall [26]. All of these features show the great potential of using transgenic plants as a bioreactor for the large scale production of cellulases and reducing the cost for enzymes through tissue autohydrolysis.

## Conclusions

With the anticipation of a fossil fuel shortage in the near future and awareness of the need for environment protection, the use of agricultural wastes and bioenergy crops for biofuel production is considered desirable. Genes coding for many cell wall degradation enzymes have been cloned and sequenced from a wide variety of microbes, and the application of plant genetic engineering in producing these enzymes for biofuel production from lignocellulose has received increasing attention and development in recent years. The most important goals of engineering plants in relation to bioenergy are to reduce the cost of biomass and cellulosic enzyme production and to produce effective cellulose decomposition enzymes on a large scale.

In this study, we adopted a transgene expression constructs that had been previously demonstrated improved *A. cellulolyticus *E1 expression in transgenic tobacco [21] for *A. cellulolyticus *E1 expression in transgenic rice, where the expression of the thermostable *A. cellulolyticus E1 *gene was under the control of a strong Mac promoter and pathogenesis related protein S signal peptide for apoplast targeting. High levels of *A. cellulolyticus *E1 protein in transgenic rice plants have been achieved via gene expression, driven by the strong Mac promoter, with accumulation in the apoplast compartment by a proper signal peptide. The bacterial gene was stably integrated into the rice genome, expressed and translated into active protein at high levels, especially in the leaf and stem, with a much higher enzyme activity than those obtained in previous studies. In the transgenic rice line with the highest expression level, the amount of *A. cellulolyticus *E1 protein accounted for 6.1% of the total soluble leaf protein with a specific activity of 79,000 pmol MU/mg protein/min. Most importantly, high levels of expression of this enzyme did not severely impair the plant growth and development of rice plants. This may be in part due to the high optimum temperatures (81°C) for the E1 activity and the relatively low plant growth temperatures (16°C to 35°C), which may not activate the E1 enzyme substantially and interfere with the cell wall integrity of transgenic plants. Furthermore, the presence of high-level active *A. cellulolyticus *E1 protein in the transgenic rice straw enhances the hydrolysis of cellulose to reducing sugars.

In the future, the advancement of plant genetic engineering can be tapped to produce biomass or crop plants with increased overall biomass and cellulose content, reduced lignin content [43] or simplified structure of hemicelluloses. It will also be interesting to simultaneously express both cellulose and hemicellulose degrading enzymes to increase the efficiency of cellulose and hemicellulose conversion into glucose and xylose, respectively. In addition, targeting the same or different foreign cellulose hydrolytic enzymes simultaneously to various cellular compartments of transgenic plants might provide the opportunity for effective production of large quantities of cellulases and increase the efficacy of tissue autohydrolysis. In short, with the advancements in modern plant genetic engineering, many cellulose hydrolysis-related genes from microbes have been cloned and introduced into plants and expressed as active proteins in large quantities. Modifying the genetic make-up for higher thermostability and protein stability during storage and for easy purification will also significantly lower the cost of cellulosic biofuel production.

## Methods

### Transformation vector and bacterial strains

The DNA fragment containing the apoplast transit peptide and mature *E1 *coding sequence from *A. hydrolyticus *(1.56 kb) was first fused in frame between the Mac promoter and nos terminator of the *p*ZD264 vector [21]. For selection of transgenic plants, *HptII *was isolated from the binary vector *p*CAMBIA1300 and inserted into the *p*ZD264 vector using the *Pme*I (GTTTAAAC) cloning site, which resulted in a vector of *p*1500 for rice transformation (Figure 1). The primers used for polymerase chain reaction (PCR) to attach *HptII *were 5'-CACACAACATACGAGCCGGAAGCAT- 3' and 5'-GCTTAGACAACTTAATAACACATTGCGGACGTT-3'. *Escherichia coli *strain DH5α was used as the host for cloning plasmid DNA while *Agrobacterium tumefaciens *AGL0 strain harboring the vector was used for rice transformation.

### Plant material, gene transformation and plant growth conditions

Immature embryos of a japonica rice cultivar (*Oryza sativa *L.), Tainoung 67 (TNG67), were used for induction of embryogenic calli. The *Agrobacterium*-mediated transformation and regeneration of transgenic plants were performed following the procedure of previous studies [44-48]. Regenerated transgenic rice plants were first cultured in a growth chamber under a day/night temperature regime of 28°C/24°C and a light period of 14 h at 400 μmol/m^2^/s. For molecular and biochemical studies, all transgenic and wild type rice pants were grown in a greenhouse under natural sun light conditions (12 h-14 h) with day temperature ranging from 25°C to 32°C and night temperature from 20°C to 25°C. Plants were watered and fertilized regularly.

### Isolation of rice genomic DNA

For the isolation of genomic DNA, leaf tissues were ground in liquid nitrogen into a fine powder using a pestle and mortar and mixed with extraction buffer containing 100 mM tris-HC1 (pH 7.5), 500 mM NaCl, 50 mM ethylenediaminetetraacetic acid (EDTA), 2% β-mercaptoethanol and 4% sodium dodecyl sulfate (SDS) [49]. The mixture was incubated at 65°C for 15 min and centrifuged (14,000 rpm) at room temperature for 3 min. The supernatant was mixed with 5 M potassium acetate and incubated at -20°C for 15 min. After centrifugation at 4°C for 20 min, the supernatant was passed through a MILLEX-HA filter (Millipore, Billerica, MA, USA; pore diameter 0.45 μm), mixed gently with an equal volume of ice-cold isopropanol, and then incubated at -20°C for 30 min, followed by centrifugation at 4°C for 20 min. The DNA pellet was washed with ethanol, air-dried and dissolved in 5 × TE buffer (50 mM tris-HCl, 10 mM EDTA, pH 8.0). One tenth volumes of 3 M sodium acetate and an equal volume of isopropanol were added and mixed well. After centrifugation at 4°C for 10 min, the DNA pellet was washed sequentially by 70% and 95% ethanol, air-dried, and then dissolved in distilled water.

### Southern blot analysis

Southern blot analysis was performed essentially as described by Sambrook *et al*. [49]. Genomic DNA was first treated with RNase A at 60°C for 2 h. Twenty-five micrograms DNA was digested by *HindI*II, and DNA fragments separated by electrophoresis on 0.8% agarose gel at 25 V for 15 h. After electrophoresis, the agarose gel was first soaked in 0.25 M HCl for 15 min for depurination, denatured by soaking in the denature solution containing 0.5 N NaOH and 1 M NaCl for 2 h, and then neutralized in 0.5 M tris-HCl (pH 7.4) and 2.5 M NaCl for 2 h. The DNA was transferred to Hybond-N+ membrane (Amersham Biosciences, Little Chalfont, Buckinghamshire) by capillary transfer using 10 × SSC buffer that contains 1.5 M sodium chloride and 0.15 M sodium citrate, pH 7.0 and subsequently fixed by UV cross-linking. Probes were prepared from the PCR products of *E1 *and *HptII *independently using genomic DNA isolated from transgenic rice plants as templates. About 200 μg of PCR products and 0.5 μg of random primers were mixed and denatured at 94°C for 3 min. Denatured PCR products were diluted to a final volume of 15 μL in Klenow buffer mixture containing 6 μg of BSA, 0.1 mM deoxyribonucleotide triphosphate without deoxycytidine triphosphate (dCTP) and 5 units of Klenow fragments of DNA polymerase I, kept at room temperature for 1 h after adding ^32^P-labeled deoxycytidine triphosphate, and then passed through a Sephadex G50 column (Sigma-Aldrich, St. Louis, MO, USA) to purify the probe. The membrane was treated with pre-hybridization solution containing 1 M NaCl, 10% sodium chloride-tris-EDTA buffer (SSTE), 1 × Denhardt's solution, 0.085% dextran sulfate and 1% salmon sperm DNA at 42°C for 4 h, and then hybridized with the probe at 42°C overnight. The hybridized membrane was washed sequentially by 0.1 × SSC containing 0.1% SDS at 42°C for 20 min, and exposed to X-ray film at -80°C for a period of time ranging from 8 h to 2 weeks, depending on radioactivity.

### RNA extraction and northern blot analysis

Total RNA was extracted according to Wang and Vodkin [50] with a modified phenol/chloroform protocol. Leaf tissues were ground in liquid nitrogen with TENS buffer, containing 80 mM tris-HCl, 16 mM EDTA, 160 mM NaCl, 4% SDS and 16 mM dithiothreitol (DTT). Reverse transcriptase-PCR was performed as described by Chen *et al*. [51] with slight modification. Total RNA (7.5 μg) was first treated with RNase-free DNase Ι by incubating in a mixture containing 6.7 mM DTT, 20 units RNAsin and 1 unit RNase-free DNase I. The mixture was mixed evenly and incubated at 37°C for 15 min and then at 80°C for 10 min [52].

RNA gel blot analysis was performed as described by Chao *et al*. [53]. Total RNA was treated with 3-(N-morpholino)propanesulfonic acid buffer, containing 5% formaldehyde and 40% formamide, and separated by electrophoresis on 1.2% agarose gel. Total RNA in the gel was stained with ethidium bromide and then treated with 50 mM NaOH for 30 min, followed by 10 × SSC for 30 min. The RNA was then transferred to a Hybond-N+ membrane by capillary transfer using 10 × SSC and then fixed onto the membrane by UV cross-linking. Probe preparation and hybridization procedures were the same as described for Southern blot analysis.

### Enzyme activity assay

Soluble protein was extracted from newly matured leaf, stem, root, green floret and mature seeds of transgenic and wild type plants by grinding in cold extracting medium containing 80 mM 2-(N-morpholino)ethanesulfonic acid (MES, pH 5.5), 10 mM β-mercaptoethanol, 10 mM EDTA, 0.1% sodium N-lauroylsarcosinate (w/v), 0.1% Triton X-100 (v/v), 1 mM phenylmethylsulfonyl fluoride (PMSF), 10 μM leupeptin, and 1 μg/mL pepsin A. The crude extract was centrifuged at 15,000 g for 10 min at 4°C and the supernatant was used immediately for enzyme assay. Endoglucanase activity was quantitated by its ability to cleave 4-methylumbelliferyl-β-D-cellobioside (MUC) to produce the fluorophore, 4-methylumbelliferone (4-MU), at 65°C [7]. The reaction was initiated by adding 50 μL enzyme extract with an equal volume of 2 × ice cold endoglucanase activity assay buffer containing 4 mM 4-MUC, 160 mM MES (pH 5.5), 20 mM β-mercaptoethanol, 2 mM EDTA, and 2 mM DTT, heated to 65°C, and terminated after 45 min by adding 0.2 M Na_2_CO_3_. The peak excitation wavelength was 365 nm and the peak fluorescence emission was 455 nm, measured with a TECAN infinite M200 fluorescence spectrophotometer (Tecan, Gordig/Salzburg, Austria). Enzyme specific activity was expressed on a total soluble protein basis.

### Antibody production

For the production of polyclonal antibodies against E1, the coding sequence of mature E1 was PCR amplified with *p*1500 as a template using a gene specific primer set (forward, 5'-AAGCTTGCGGGCGGCGGCTATTGGCACACGA-3'; reverse, 5'-CTCGAGTGTCGGTGCCGCGTTGCTTCCGGTA-3') with *Hind*III/*Xho*I restriction sites. The resulting PCR product was digested with *Hind*III and *Xho*I, and inserted into the prokaryote His-tag expression vector *p*QEtrisystem (Invitrogen, Valencia, CA, USA) to construct the *p*TRI/E1 vector. *E. coli *strain BL21 was used for the production of recombinant protein, and overexpression of the truncated protein was induced according to the manufacturer's instructions. The E1 protein from the sonicated cell extract was primarily purified by TALON metal affinity resin (Clontech, Mountain View, CA, USA) and further separated by electrophoresis on 10% SDS-PAGE. Rabbits were immunized with 100 μg of protein in the first injection with four booster injections at one-week intervals, each with 50 μg of protein. After clearance by centrifugation, the crude serum obtained from the rabbits was used for immunological studies. For antibody titer determination, purified E1protein was separated on 10% SDS gels and blotted onto polyvinylidene fluoride (PVDF) membranes (Bio-Rad, Hercules, CA, USA) with electrophoresis. The blots were incubated with the antibody with a series of dilution, and immunodetection was carried out using alkaline phosphatase conjugated goat anti-rabbit immunoglobulin G.

### SDS-PAGE, western blot and zymography

The total soluble protein was extracted from newly matured leaves by the same method as described above for enzyme assay. The protein concentration was determined using Quick Start Bradford Protein Assay (Bio-Rad, Hercules, CA, USA) with BSA as standard according to the instruction manual. The lanes were loaded with 20 μg of proteins per lane was loaded onto 10% polyacrylamide gel and separated by electrophoresis [54]. The protein on the gel was transferred on to PVDF membrane (Bio-Rad, Hercules, CA, USA) with electrophoresis, and probed with rabbit antibodies against purified E1 (1:100,000 dilution) and tobacco Rubisco large subunit independently. Goat anti-rabbit immunoglobulin G conjugated to alkaline phosphatase was used as a second antibody. Color formation was developed by adding 5-bromo-4-chloro-3'-indolyphosphate p-toluidine salt/nitro-blue tetrazolium chloride (BCIP/NBT) substrate solution (Sigma-Aldrich, St. Louis, MO, USA) directly onto the membrane at room temperature.

The assay of E1 activity by a zymogram was performed with leaf soluble protein separated in 10% polyacrylamide gel containing 0.1% CMC according to Chavez *et al*. [55] with minor modifications. After electrophoresis, the gel was washed with 50 mM phosphate-buffered saline (pH 5.3) for 30 min, 2.5% triton X-100 for 4 h and incubated at 65°C in 50 mM phosphate-buffered saline for 30 min. The gel was stained in 1% Congo red solution for 15 min at room temperature, and then washed with 1 M NaCl to enable visualization of cleared zones, which correspond to the E1 endoglucanase activity. The amount of E1 protein accumulated in the leaves among transgenic plants was estimated with QuantOne software v4.3.0 (BioRad, Hercules, CA, USA) based on the densitometric determination from the zymogram, where the purified E1 protein at 1 μg, 2 μg and 3 μg per lane were included as standards.

### Hydrolysis of rice straw with cultured CGF

Fresh CGF was collected from a local abattoir, quickly strained through four layers of cheesecloth, mixed with AS in a 1:5 ratio (v/v) and incubated statically at 39°C in flasks under anaerobic condition by flushing with CO_2 _gas. The AS was based on McDougall buffer [56], which contained 117 mM NaHCO_3_, 26 mM Na_2_HPO_4 _· 12H_2_O, 8 mM NaCl, 8 mM KCl, 0.2 mM CaCl_2_, 0.3 mM MgCl_2 _(pH 8.1). The AS also contained 2% napier grass powder as the sole carbon source. A repeated-batch culture was conducted. After 2 days of incubation, the culture medium was centrifuged (3,200 *g*, 30 min) and the supernatant was used as inoculums for the next culture. The supernatant of the fifth repeated-batch culture was collected, concentrated five times and stored at -20°C until use. The activities of xylanase, cellulase and avicelase in the concentrated supernatant were analyzed based on the amount of liberated reducing sugars by the 3,5-dinitrosalicylic acid method [57-60]. The concentrated supernatant contained approximately 18 U xylanase per mL, and no cellulase activity was detected. The 200-μL reaction mixture contained the same amount of the concentrated supernatant in buffer pH 8.0 and 1% (w/v) of the corresponding substrates: birch wood xylan for xylanase activity, CMC for CMCase activity, and avicel for avicelase activity. The reactions were incubated at 39°C for 10 min and the amount of reducing sugar was determined by absorbance measurements based on standard curves prepared using the corresponding sugars. One unit was defined as the amount of enzyme liberating 1 μmol of reducing sugar, or the corresponding products, per minute under the assay conditions.

For enzymatic hydrolysis of the rice straw, rice straw was harvested without grains at mature stage and dried in air. The entire straw was milled into fine powder (passed through a 1 mm mesh), and 0.1 g straw sample was mixed with 10 mL AS or 10 mL concentrated supernatant of the cultured CGF, and incubated at 39°C for 1 h, 39°C for 2 h, or 39°C for 1 h plus 81°C for 1 h. Each experiment was repeated three times. The amount of sugars released during 1 h and 2 h incubations was determined using a 3,5-dinitrosalicylic acid method, with D-glucose as standard. A Mixed Sucrose-D-fructose- D-glucose Assay Kit (Megazyme, Wicklow, Ireland) was used for quantification of free glucose, sucrose and fructose, according to the manufacture's instructions.

## Abbreviations

AS: artificial saliva; BSA: bovine serum albumin; CGF: cow gastric fluid; CMC: carboxymethyl cellulose; DTT: dithiothreitol; E1: cellulose hydrolytic enzyme β-1, 4-endoglucanase; EDTA: ethylenediaminetetraacetic acid; kDa: kiloDaltons; MES: 2-(N-morpholino)ethanesulfonic acid; MUC: 4-methylumbelliferyl-β-D-cellobioside; PVDF: polyvinylidene fluoride; saline-sodium citrate.

## Competing interests

The authors declare that they have no competing interests.

## Authors' contributions

HLC conducted the rice transformation and characterized the molecular and biochemical properties of transgenic rice. HLC and MSBK designed the experiments, analyzed the data and wrote the paper. CWH performed enzymatic hydrolysis of rice straw by cow gastric fluid. ZD constructed the transgene expression vector, analyzed the results and reviewed the manuscript.
